# Antioxidant and Antiaging Properties of a Novel Synergistic Nutraceutical Complex: Readouts from an In Cellulo Study and an In Vivo Prospective, Randomized Trial

**DOI:** 10.3390/antiox11030468

**Published:** 2022-02-26

**Authors:** Sophia Athanasopoulou, Marianna Kapetanou, Michel Georges Magouritsas, Nikoletta Mougkolia, Polykseni Taouxidou, Michael Papacharalambous, Fotios Sakellaridis, Efstathios Gonos

**Affiliations:** 1 Institute of Chemical Biology, National Hellenic Research Foundation, 11635 Athens, Greece; sathan@eie.gr (S.A.); mkapetanou@eie.gr (M.K.); n.mougkolia@pasteur.gr (N.M.); 2 Faculty of Medicine, School of Health Sciences, University of Thessaly, 41334 Larisa, Greece; 3 TheraCell Advanced Biotechnologies, 14564 Kifisia, Greece; mgmagouritsas@apphialabs.com (M.G.M.); fotis.sakellaridis@qualiapharma.com (F.S.); 4 Department of Physical Education and Sport Science, Aristotle University, 57001 Thessaloniki, Greece; xeniataou@gmail.com; 5 Orthobiotiki Clinic, 15125 Marousi, Greece; mpapa@orthobiotiki.com; 6 Metropolitan Hospital, 18547 Athens, Greece; 7 Hellenic Pasteur Institute, 11521 Athens, Greece

**Keywords:** nutraceuticals, dietary supplements, antioxidant defense, proteostasis, well-being

## Abstract

Aging is a dynamic procedure that is developed in multiple layers and characterized by distinct hallmarks. The use of biomarkers that target different hallmarks of aging is substantial in predicting adverse outcomes during the aging process, implementing specifically designed antiaging interventions and monitoring responses to these interventions. The present study aimed to develop a novel composition of plant extracts, comprising identified active ingredients that synergistically target different hallmarks of aging in cellulo and in vivo. The selected single extracts and the developed composition were tested through a powerful set of biomarkers that we have previously identified and studied. The composition of selected extracts simultaneously increased cellular lifespan, reduced the cellular oxidative load and enhanced antioxidant defense mechanisms by increasing proteasome activity and content. In addition, the combination prevented telomere attrition and preserved optimum DNA methylation levels. Remarkably, biomarker profiling of healthy volunteers who received the identified combination in the form of a nutritional supplement within the frame of a prospective, randomized, controlled 3-month trial revealed an unprecedented antioxidant capacity in humans. In conclusion, our results support the notion that interventions with specifically designed combinations of natural compounds targeting multiple hallmarks of aging represent an effective way to improve healthspan and well-being.

## 1. Introduction

Human aging is characterized by a progressive decline in organismal function, which is also the main risk factor for prevalent age-related diseases, such as neurodegenerative disorders, diabetes and cancer [1]. The core underlying aging mechanisms have been described as the “Hallmarks of Aging” [1]. Genomic instability, telomere attrition, epigenetic alterations and loss of proteostasis represent some primary hallmarks that cause damage to cellular functions. Likewise, deregulated nutrient sensing and cellular senescence are secondary aging hallmarks, which confer antagonistic responses to such damage. Comprehensive studies of these mechanisms and their crosstalk have led to the discovery of new biomarkers and novel therapeutic targets of antiaging interventions, and they have demonstrated that environmental and genetic interventions can ameliorate the manifestation of aging [2].

The proteostasis network and its key regulatory component—the proteasome—represent a primary hallmark of aging and thus an efficient cellular target of antiaging interventions. Specifically, the proteasome is a multi-subunit protease complex, composed of the catalytic 20S “core” and the regulatory 19S “lid” that selectively catalyze the degradation of short-lived regulatory proteins and oxidized or damaged peptides [3,4]. Previous studies from our laboratory have established the inverse relationship between proteasome levels and oxidized proteins, both in vitro and in vivo [5,6,7]. Moreover, we have shown that a functional proteasome is linked to the extreme longevity seen in centenarian populations [8] and is associated with reduced frailty in the elderly [6]. Concomitantly, proteasome activation, either genetically or with natural compounds, enhances the lifespan of human cells [9], enhances stemness [10], alleviates the pathological phenotype of protein aggregation-related diseases and improves lifespan of model organisms [11,12]. In support of the eminent role of proteostasis in multiple aspects of cellular responses to environmental stimuli, we have established the regulatory role of the transcription factor FoxO1 on proteasome activity [13].

Telomeres’ dynamics are another major pillar of cellular homeostasis that is largely affected by exposure to stressors. Systematic telomere attrition may threaten genomic integrity and contribute to aging, as well as to many age-related diseases [14,15]. Importantly, accumulating evidence, also including studies from our laboratory, has demonstrated the shortened leukocyte telomere length (LTL) in humans is not only primarily associated with age [1,16,17,18] and metabolic status [16,19,20], but also with exposure to chronic psychological stress [21] and depression [22], and it represents a risk factor for a range of chronic pathologies, such as diabetes [23,24]. In vitro studies have shown that enzymes that modulate cellular metabolic status, such as SIRT1, act as positive regulators of telomere length and attenuate age-related telomere shortening [25]. Hence, cellular telomeres status can change in response to such epigenetic mechanisms depending on lifestyle factors [26,27], representing a key target of innovative interventions that may positively influence healthspan.

Epigenetic modifications are important regulators of gene expression that can be easily altered by both aging and dietary interventions. DNA methylation primarily occurs in CpG (5′- C- phosphate—G- 3′) dinucleotides and is generally associated with transcriptional repression [28]. During aging, DNA methylation patterns are characterized by alterations in 5-methylcytosine (5meC) content. The age-associated drift of methylation patterns includes both hyper- and hypo-methylation at specific genomic regions that differ between tissues [29,30]. Based on the methylation state of specific CpGs islands in the genome, new tools have been developed to estimate the epigenetic age and conditions that contribute to epigenetic aging acceleration [31]. These changes in the patterns per se represent potential aging or disease biomarkers and, therefore, effective targets for therapeutic or dietary interventions.

Simultaneous modulation of the described mechanisms of the aging process may be needed to optimize effector outputs and, hence, health benefits. Here, we identify a novel composition of plant extracts with powerful effects in multiple hallmarks of cellular senescence and antioxidant defense. Remarkably, biomarker profiling of healthy volunteers who received the identified combination in the form of a nutritional supplement within the frame of a prospective, randomized, controlled trial, revealed an unprecedented antioxidant capacity in humans.

## 2. Materials and Methods

### 2.1. In Cellulo Evaluation

#### 2.1.1. Cell Cultures

HFL-1 human diploid fibroblasts (HDFs) were used in in vitro conditions (growth at 37 °C, 5% CO_2_ and 95% humidity). The cells were maintained in Dulbecco’s modified Eagle’s medium (Thermo Fisher Scientific, Waltham, MA, USA) supplemented with 10% (*v*/*v*) fetal calf serum (Thermo Fisher Scientific, Waltham, MA, USA), 100 Units/mL penicillin, 100 μg/mL streptomycin, 2 mΜ glutamine and 1% (*v*/*v*) non-essential amino acids (complete medium). The cells were constantly cultured in a culture medium supplemented with the composition (denoted as “HS” in all Figures and Tables) in two concentrations: 2 and 5 μg/mL, diluted in DMSO (dimethyl sulfoxide). The control cultures were incubated in a medium supplemented with 0.1% DMSO.

The cells were replenished with fresh media supplemented with the composition or the diluent every 72 h, and their numbers were examined using a Coulter Z2 counter (Beckman Coulter, Brea, CA, USA) until they reached senescence (approximately after 13 weeks). The media were replenished 16h before every assay. The cumulative population doublings performed by each culture were calculated using the following formula: CPD = Σ(PD) where PD = LOG(Nfinal/Ninitial)/LOG [2], where N final represents the number of cells that were measured when each culture reached confluence, and N initial represents the number of cells that were initially seeded.

#### 2.1.2. Real-Time PCR Analysis

##### Transcriptional Levels of Proteasome Subunits

Following incubation with the composition during their lifespan, cells were collected, lysed, and total RNA was isolated using TRIzol and was transcripted into cDNA with the cDNA iScript synthesis kit (Bio-Rad Laboratories, Hercules, CA, USA). The Real-Time PCR was performed in triplicate using the CFX Connect Real-Time PCR Detection System. The Real-Time PCR primer sequences are summarized in Supplementary Table S1. Quantitative RT-PCR was performed using the iQ SYBR Green Supermix (Bio-Rad Laboratories, Hercules, California Laboratories, Hercules, CA, USA). Relative quantities of transcripts were determined using the relative standard curve method normalized to GAPDH with the iCycler iQ software Gene Expression MacroTMversion1.1 (Bio-Rad Laboratories, Hercules, CA, USA).

##### Relative Telomere Length

Cells were collected following treatment with the composition throughout their lifespan, and DNA extraction was performed with the NUCLEOSPIN extraction kit (Macherey-Nagel, Düren, Germany). DNA concentration was determined by measuring the absorbance at 260nm with a UV-VIS spectrophotometer and multiplying it by the dilution factor. Relative Telomere Length was analyzed by quantitative PCR, according to Cawthon’s protocol [32], using CFX Connect Real-Time PCR Detection System (Bio-Rad Laboratories, Hercules, California Laboratories, Hercules, CA, USA). The relative telomere (T) lengths (T/S ratios) were analyzed by monochrome multiplex quantitative PCR (MMQPCR), using a single-copy gene amplicon primer set for albumin (S) and a telomere-specific amplicon primer set (T).

#### 2.1.3. Immunoblot Analysis

##### Oxidized Protein Levels

Cells were treated with the composition short term (24 h) and long term every 72 h during their replicative lifespan, and the media were replenished 16 h before cell lysis and protein extraction. The extracted proteins (10 μg) were subjected to SDS-PAGE (sodium dodecyl sulfate-polyacrylamide gel electrophorese), using 10% Mini-PROTEAN TGX Stain-Free Precast Gels (Bio-Rad Laboratories, Hercules, California Laboratories, Hercules, CA, USA), followed by OxyBlot analysis (S7150, Sigma-Aldrich, St. Louis, MO, USA) that detects the carbonylated groups of proteins. Densitometry analysis for the quantification of immunoblots was performed with Bio-Rad’s Image Lab software 6.0.1. GAPDH (ab9485, Abcam, Cambridge, UK), which was used as a control for equal protein loading.

##### Proteasome Subunits

Twenty μg of protein from the cell lysate from harvested HFL-1 fibroblasts was mixed with non-reducing Laemmli buffer and separated by SDS-PAGE using 10% Mini-PROTEAN TGX Stain-Free Precast Gels (Bio-Rad Laboratories, Hercules, California Laboratories, Hercules, CA, USA). Following electrophoresis, the total protein load was quantified using the StainfreeTM technology, and the proteins were transferred to nitrocellulose membrane to be probed for β5 (BML-PW8895, Enzo Life Sciences, Farmingdale, NY, USA), α7 (BML-PW8110, Enzo Life Sciences, Farmingdale, NY, USA) and Rpt-6 (BML-PW8215, Enzo Life Sciences, Farmingdale, New York, NY, USA). The secondary antibodies (ab205718; ab6728, Abcam, Cambridge, UK) were conjugated with horseradish peroxidase and detected the bound primary antibody by enhanced chemiluminescence using the Chemidoc XRS + imaging system (Bio-Rad Laboratories, Hercules, California Laboratories, Hercules, CA, USA).

#### 2.1.4. DNA Methylation Levels

DNA extraction was performed in collected cells after treatment with the NUCLEOSPIN extraction kit. DNA concentration was determined as described in the Real-Time PCR analysis. The measurement of global DNA methylation status was performed by using the Global DNA methylation kit (ab117128, Abcam, Cambridge, UK), and for each sample, the input for the assay consisted of 100 ng of isolated genomic DNA, in technical duplicates. Absorbance was read at 450 nm using a Safire II microplate reader (TECAN, Männedorf, Switzerland), and Global DNA Methylation was calculated as the percentage of methylated DNA (5-mC) in total DNA through the generation of a standard curve and the relevant formula.

#### 2.1.5. Enzymatic Activity Assays

##### Foxo1 Transcriptional Activity Assay

The isolation of nuclear proteins and the subsequent examination of FOXO1 transcriptional activity were performed with the Nuclear Extraction Kit (ab113474, Abcam, Cambridge, UK) and the FOXO1 Transcription Factor Assay Kit (Colorimetric; ab207204, Abcam, Cambridge, UK), respectively, according to manufacturer’s instructions.

##### SIRT1 Activity Assay

SIRT1 activity was determined with the SIRT1 Activity Assay Kit (Fluorometric) (ab156065, Abcam, Cambridge, UK) that allows the rapid and sensitive evaluation of SIRT1 inhibitors or activators using purified SIRT1 or detects SIRT1 activity in lysates, according to the manufacturer’s instructions.

##### Proteasome Activity Assay

Harvested cells after the short-term (24 h) and long-term treatment with the indicated compounds were lysed in 25 mM Tris/HCl lysis buffer, pH 7.6 containing 5 mM ATP, 10% glycerol, 20 mM KCL, 1 mM EDTA, 1 mM DTT, 0.2% Nonidet P-40, 10 mM phenylmethylsulfonyl fluoride and 10 μg/mL aprotinin. CT-L activity of the proteasome was determined after incubating 10 μg of total protein for 30 min at 37 °C with the fluorogenic peptide LLVY-AMC (Enzo Life Sciences, Farmingdale, NY, USA), as previously described [33]. Fluorescence was measured using the Safire II plate fluorescence spectrophotometer (TECAN, Männedorf, Switzerland). In cells or tissue samples, proteasomal activity was determined as the difference between total activity and activity in the presence of 20 μΜ of the proteasome inhibitor MG132 (Enzo Life Sciences, Farmingdale, NY, USA). Protein concentration was determined by Bradford Protein Assay (Bio-Rad Laboratories, Hercules, CA, USA) using bovine serum albumin as standard.

### 2.2. In Vivo Evaluation

#### 2.2.1. Clinical Trial Design

The study was designed as a randomized, placebo-controlled trial with follow-up at 3 months (ClinicalTrials.gov Identifier: NCT05202652). All subjects gave their informed consent for inclusion before they participated in the study. The study was conducted in accordance with the Declaration of Helsinki, and the protocol was approved by the Bioethical Committee of the National Hellenic Research Foundation (Protocol number 2515/31.5.2019), where the study was conducted. Inclusion criteria for the participation in the study were (i) residence in the Athens metropolitan area and (ii) age 29–85. Exclusion criteria were (i) diagnosed malignant disease, subjects who were under chemotherapy, therapy with biological factors and radiotherapy, (ii) use of nutritional supplements during the clinical trial period, (iii) diagnosed autoimmune diseases or other chronic diseases and (iv) subjects who had lived less than 50% of their life in the country that is their current residence. Questionnaires were created according to previous clinical trials that were conducted in our laboratory within the framework of European research programs [34,35,36]. Recruitment started on 1 September 2019 and ended on 30 September 2020. For each participant, the follow-up was 3 months after the enrolment. All the study participants received informative material, filled a questionnaire regarding the self-assessment of their health status and nutritional habits, and signed their written informed consent. Compliance was monitored by telephone or via emails. The allocation ratio in the study design was 2:1 for the intervention’s arm with subjects who received capsules with active compounds versus the placebo arm, to avoid loss of power from drop-out in this specific subgroup. In total, excluding the participants that did not return for the follow-up (dropouts), 122 apparently healthy volunteers 29–85 years of age were followed. Excluding dropouts, analysis was conducted in 43 of them that were randomly assigned in the placebo subgroup and 79 receiving the composition with the active ingredients ( Supplementary Figure S2—Consort Flow Diagram). Research works that performed the experiments and assessed the outcomes were blinded, as the assignments for intervention, allocation and randomization were performed by a healthcare practitioner, who was the person responsible for recruitment and also collected the blood samples from each subject. Blinding codes were kept on a personal computer, locked in the office that was used only by the person responsible for the study.

#### 2.2.2. Blood Handling, PBMCs and Plasma Isolation

Blood samples were collected in ethylenediaminetetraacetic acid (EDTA) vials (Sarstedt, Nümbrecht, Germany) using a sterile technique. Centrifugation at 2000× *g* for 15 min at 4 °C was performed to separate and collect the plasma that was stored in −80 °C. The remaining blood was used to isolate PBMCs according to Boyum methodology (1968), which is based on gradient density. Tubes with blood were diluted 5 times with PBS and layered on Percoll Solution (GE Healthcare, Chicago, IL, USA) and were centrifuged at 400× *g* for 20 min at 20 °C, stopping without brake; PBMCs layer was recovered and washed with cold PBS by two centrifugation steps at 600× *g* for 10 min at 4 °C. In the last centrifugation step cells were re-suspended, divided in separate tubes and stored at −80 °C for subsequent DNA assays (telomere length and methylation analyses (as described above) and immunoenzymatic analysis of the proteasome content.

#### 2.2.3. Immunoenzymatic Assays

##### Determination of 20S Proteasome Content

Total protein lysates were obtained from PBMCs, and protein content was determined by Bradford protein assay (Bio-Rad Laboratories, Hercules, California Laboratories, Hercules, CA, USA). The determination of 20S Proteasome content was performed in protein lysates as described previously [6], using 20S Proteasome ELISA Kit (BML-PW0575, Enzo Life Sciences, Farmingdale, NY, USA). Optical density was read at 450 nm on the Safire II microplate reader (TECAN, Männedorf, Switzerland).

##### Oxidized Proteins in Human Plasma

Carbonyl groups of proteins were detected using the “Protein Carbonyl ELISA Kit” (ab238536; Abcam, Cambridge, UK). Measurements were performed in duplicates, and cell lysate samples were diluted 250-fold to 10 μg/mL protein with PBS before adsorption onto 96-well Protein Binding ELISA Plates. Absorbance was measured using the Safire II microplate reader (TECAN, Männedorf, Switzerland), and the protein carbonyl content in unknown samples was determined using the standard curve derived from the BSA standards.

### 2.3. Statistical Analysis

Statistical analysis was performed using Prism Graph Pad 5.0 Software. Significance was taken as follows: *p*-values < 0.05 (*), *p*-values < 0.01 (**), *p*-values < 0.001 (***). Positive or negative correlations between the aforementioned biomarkers within the same timeframe (T0—baseline values for subjects before the intervention, or T1—values for subjects post-intervention) were determined in the total of samples using Spearman’s correlation coefficient (r). Biomarkers from T0 samples along with corresponding values from T1 samples for distinct subgroups were analyzed by two-tailed Student’s paired t-test and Wilcoxon’s paired t-test. Linear regression analysis was used to determine the relationship between different statistical variables.

## 3. Results

### 3.1. Formulation of the Administered Nutritional Supplement

**Figure 1:**
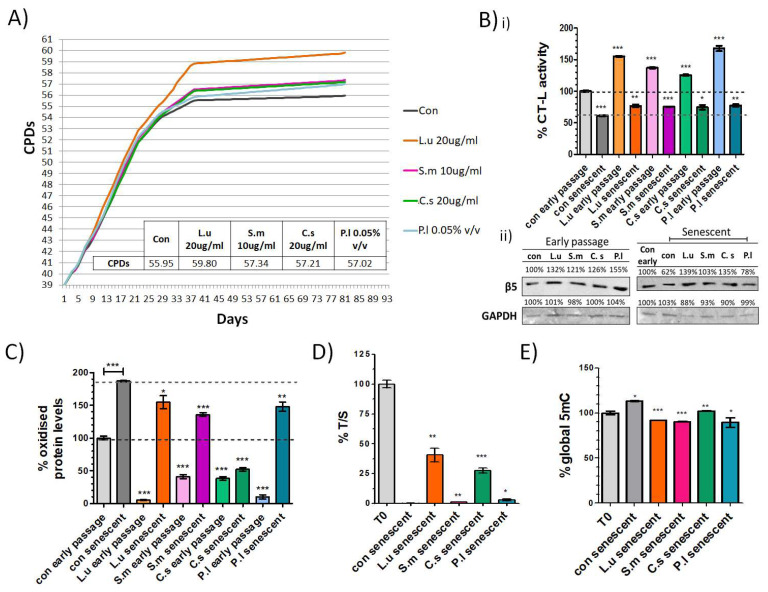
The formulation extends cellular replicative lifespan and reduces the manifestation of aging biomarkers. (**A**) Number of cumulative population doublings (CPDs) of human embryonic fibroblasts continuously treated with the indicated extracts or the solvent (control) as a function of time in culture. (**B**) (**i**) % CT-L activities and (**ii**) immunoblot analysis of the catalytic β5 proteasome subunit, analysis of (**C**) oxidized protein levels, (**D**) the indicative telomere length T/S ratio and (**E**) global DNA methylation levels in early passage and terminally senescent human fibroblasts grown in media continuously supplemented with the indicated extracts or the solvent (control), throughout their lifespan. The signal in early-passage/T0 control cells was arbitrarily set to 100% in all described assays. GAPDH was used as a control for equal protein loading. ***: *p* < 0.001, **: *p* < 0.01, *: *p* < 0.05. L.u: *Linum usitatissimum*, S.m: *Silybum marianum*, S.c: *Cynara scolymus, P.l:* *Pistacia lentiscus*.

The present formulation was developed using a library of extracts and compounds that were previously screened in the National Hellenic Research Foundation (NHRF) for their potential effect as proteasome activators that significantly decrease the oxidative load in treated cells (HFL-1, WJ MSCs and other cell lines; data not shown). This screening analysis indicated the four most potent extracts and their optimized concentrations, namely *Linum usitatissimum* (20 μg/mL), *Silybum marianum* (10 μg/mL), *Cynara scolymus* (20 μg/mL) and *Pistacia lentiscus* (0.05% *v*/*v*), that were further tested for their ability to delay senescence and the manifestation of relevant biomarkers. All four extracts enhanced the replicative lifespan of primary human fibroblasts. Specifically, cells treated continuously with *Linum usitatissimum*, *Silybum marianum, Cynara scolymus* or *Pistacia lentiscus* exhibited a 6.9%, 2.5%, 2.3% and 1.9% increase in cellular lifespan in comparison to diluent control (DMSO), respectively (Figure 1A). All four extracts significantly enhanced proteasome CT-L activity in early-passage cells (155.0%, 137.0%, 125.3% and 167.7%, respectively), while continuous treatment with the selected extracts partially rescued the senescence-related decrease in proteasome activity (Figure 1B(i)). The observed enhancement of CT-L activity was correlated to an increase in protein levels of the catalytic β5 proteasome subunit (Figure 1B(ii)). Moreover, the improved proteasome activity in both early- and late-passage cells treated with the selected extracts eliminated the load of oxidatively modified proteins (Figure 1C). Additionally, the degree of telomere shortening when the cells entered senescence was markedly reduced in cells treated with *Linum usitatissimum* or *Cynara scolymus* (maintained at 40.6% and 27.6% of the initial T/S ratio, respectively, in contrast to 0.2% that control senescent cells exhibited; Figure 1D). Finally, we detected a senescence-related increase (+13.2%) in global DNA methylation in fibroblasts treated with the diluent that was rescued in cultures that were continuously treated with the selected extracts (Figure 1E). In summary, the four extracts positively affected all assayed biomarkers.

Based on the results described in Figure 1, we combined the four extracts and active ingredients described in detail in Table 1 and Supplementary Table S2 in mixtures of different concentrations and ratios to evaluate potentially synergistic or antagonistic effects and to identify the most effective combination (Supplementary Figure S1). Extract Combination No. 3, as assessed in Supplementary Figure S1A, was the combination demonstrating the highest scores for each assay. Based on these assays, we developed the formulation described in Table 1. This formulation (referred to as the HS composition) was used in subsequent experiments for the in cellulo assessment of its antiaging and antioxidant capacity.

### 3.2. Antiaging and Antioxidant Properties of the Composition Assessed In Cellulo

#### 3.2.1. The Composition Extends the Lifespan of Human Diploid Fibroblasts In Vitro

**Figure 2:**
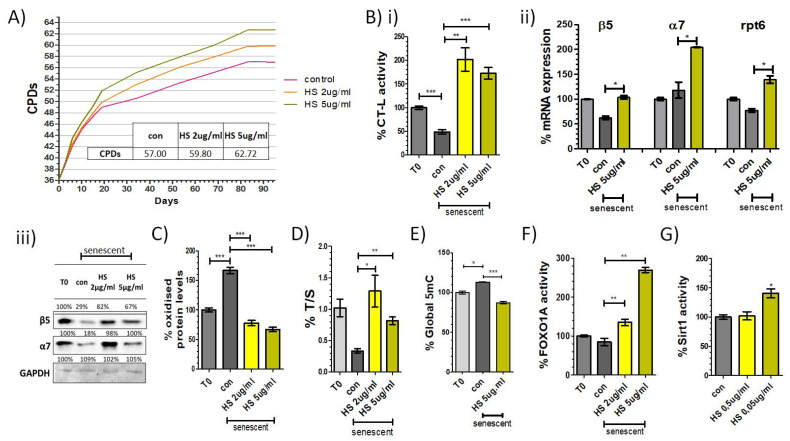
Antiaging and antioxidant properties of the composition in human primary cell cultures: (**A**) Number of cumulative population doublings (CPDs) of human embryonic fibroblasts continuously treated with the indicated composition concentrations or the diluent DMSO 0.1% (control) as a function of time in culture. (**B**) (**i**) % CT-L activities, (**ii**) mRNA levels of β5, α7 and Rpt6 proteasome subunits, (**iii**) immunoblot analysis of the catalytic β5 proteasome subunit and of the structural core α7 subunit. (**C**) oxidized protein levels, (**D**) analysis of the indicative telomere length T/S ratio, (**E**) global DNA methylation levels, (**F**) FoxO1 transcriptional activity and (**G**) Sirtuin1 direct activation and in early passage (T0) and terminally senescent human fibroblasts grown in media continuously supplemented with the composition concentrations or the diluent DMSO 0.1% (control), throughout their lifespan. The signal in early-passage (T0) control cells was arbitrarily set to 100% in all described assays. GAPDH was used as a control for equal protein loading. ***: *p* < 0.001, **: *p* < 0.01, *: *p* < 0.05.

First, we examined the replicative lifespan of human embryonic fibroblasts incubated with the HS composition in concentrations of 2 and 5 μg/mL and the diluent DMSO 0.1% as a control. Cells that were treated with the composition in concentration 2 μg/mL or 5 μg/mL displayed a 4.9% or 10.0% extension of replicative lifespan, respectively, as compared to their control counterparts (Figure 2A). This demonstrates the increased effectiveness of our composition in delaying replicative senescence.

#### 3.2.2. The Composition Increases the Proteasome Content and Activity and Diminishes the Levels of Oxidized Proteins

Following the intriguing finding that HS confers lifespan extension in the treated cells, next, we examined this effect from the prospect of proteostasis. As an enhanced proteasome activity associates positively with longevity and mainly the elimination of protein aggregates, we examined the composition’s effect on the proteasome during replicative senescence. Early-passage (T0), senescent control cells (DMSO) and senescent cells were treated with the composition in concentrations of 2 and 5 μg/mL (HS) or the diluent DMSO. As shown in Figure 2B(i), CT-L activity was induced by 415.3% in the presence of the composition in concentration of 2 μg/mL and by 354.8% in the presence of the composition in concentration of 5 μg/mL, as compared to the activity detected in their relative control cultures. Concomitantly, cells supplemented with the composition in concentration of 5 μg/mL demonstrated the optimal induction of mRNA expression levels of the core catalytic (β5, 165.6% increase) and structural (α7, 173.3% increase) proteasome subunits and of the 19S proteasome regulatory subunit rpt6 (180.0% increase; Figure 2B(ii)). In support to these findings, the composition rescued the senescence-related decline of protein levels of β5 and α7 proteasome subunits (Figure 2B(iii)). The increased expression levels of the aforementioned proteasome subunits are consistent with increased proteasome activities recorded in these cells. Moreover, cells treated with the composition in concentrations of 2 and 5 μg/mL (HS) exhibited a 214.1% and 249.2% reduction in oxidized proteins, respectively, as compared to their relative control cultures (Figure 2C). Thus, our data support a major role of the composition in preventing the senescence-related loss of proteostasis.

#### 3.2.3. The Composition Attenuates Age-Related Telomere Attrition

To further investigate the antiaging effects of the HS composition, we focused on another primary hallmark of aging and also a major pillar of cellular homeostasis, which is telomeres’ dynamics. An increased relative telomere length expressed as T/S ratio was observed in senescent cells treated with the composition in concentrations of 2 and 5 μg/mL (128.7% and 81.7%, respectively, of the starting T/S ratio), in comparison with their relative senescent control cultures (33.6% of the starting T/S ratio). Thus, continuous treatment of HFL-1 HDFs with the composition protected the cells from telomere shortening after extensive cellular proliferation (Figure 2D).

#### 3.2.4. The Composition Protects from Age-Related Changes in DNA Methylation

To gain further insight into the bioactivity of the HS composition with regard to epigenetic alterations in the cell, DNA methylation profiles were also evaluated. As shown in (Figure 2E), senescent cells treated with the composition in a concentration of 5 μg/mL exhibited lower global DNA methylation levels, in a profile similar to that of early-passage cells. Specifically, treatment of HFL-1 HDFs with the composition throughout their replicative lifespan prevented the 13.2% increase in global DNA methylation that control senescent cells exhibited.

#### 3.2.5. The Composition Enhances FoxO1 Transcriptional Activity

Following the detected positive effect of the composition on proteasome expression and activity, next, we explored potential mechanistic evidence that may hint at its transcriptional regulation. To this end, we assessed the effects of the composition on FoxO1, a transcription factor that links nutrient sensing cues to longevity. The binding of FoxO1 to its target sequences was significantly improved in cells treated with the composition, as compared to the levels in their control counterparts (Figure 2F). The optimal FoxO1 activation (269.4%) was achieved with the composition in concentrations of 5 μg/mL. The proteostasis-promoting effects of the composition may, therefore, result, at least partially, through an enhancement of FoxO1 activity.

#### 3.2.6. The Composition Activates SIRT1

Finally, to examine potential underlying mechanisms of the positive effects of the HS composition on the attenuation of telomere attrition and cellular antioxidant capacity, we tested the direct activation of SIRT1, a NAD+-dependent histone deacetylase (HDAC) proposed to contribute to genomic integrity via positive regulation of telomere length and antioxidant activity. Our results demonstrated that the composition directly activated SIRT1 (+40.5%) at a concentration of 0.05 μg/mL (Figure 2G). These data suggest that the composition might also exert anti-senescence effects via SIRT1 activation.

### 3.3. Antiaging, Antioxidant and Well-Being Properties Assessed In Vivo

**Figure 3:**
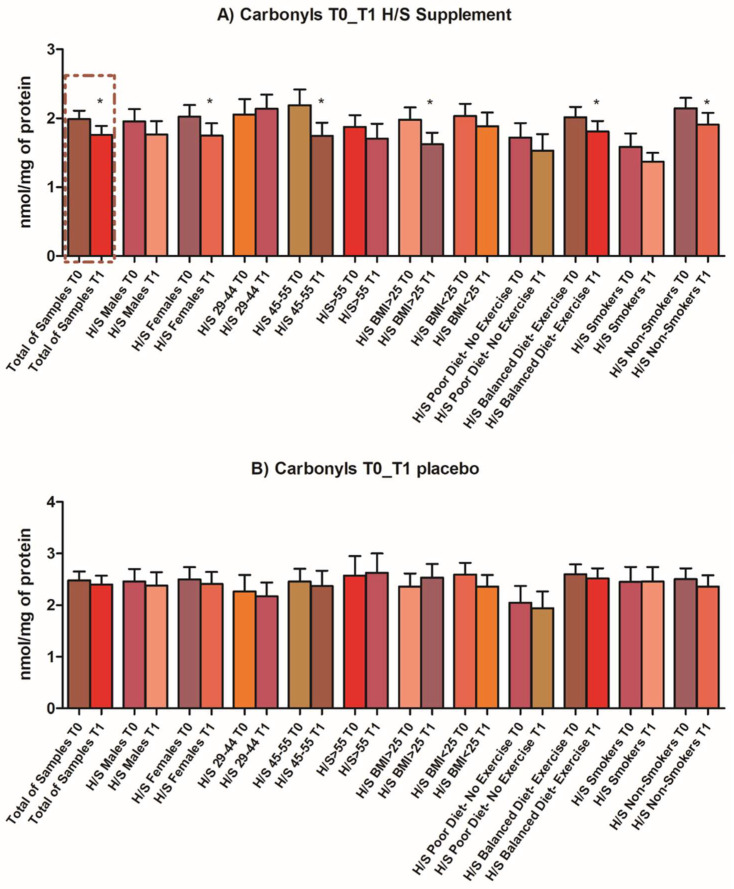
Antioxidant effects assessed in vivo: Levels of oxidized proteins pre- (T0) and post-intervention (T1) in the total sample (paired t-test, *p*-value 0.0175), as well as in distinct subgroups, adjusted for sex, age, BMI, dietary status/exercise and smoking for the intervention arm that received the capsule with active ingredients and the arm that received the placebo. The biomarker was tested in isolated human plasma. * Statistical significance between values before and after the intervention, *p* < 0.05.

Following the reported intriguing in vitro findings to determine whether the identified composition could exert important beneficial effects when incorporated into human diet, the composition and the placebo were administered in healthy volunteers aged 29–85 years, in the form of an oral capsule, for three consecutive months. The capsule content is described in detail in Table 1. Chemical characterization of the selected extracts is summarized in Supplementary Table S2. The primary outcome of the clinical in vivo study was the statistically significant decrease in oxidized plasma proteins for the intervention’s arm that received the composition with active ingredients, while for the placebo subgroup, no changes in oxidation status were observed. No harm or unintended effects were recorded. Adjustment for distinct subgroups according to the questionnaires revealed that, while the trend of decrease was common in every subgroup, female volunteers, participants aged 45–55 and with a BMI > 25, non-smokers and subjects who followed a balanced diet and exercise were those exhibiting the greater difference in levels of oxidized proteins pre- and post-intervention (Figure 3). All biomarkers were assayed before and after the intervention in both arms, but profound differences following the intervention were observed in oxidized protein proteolysis.

**Figure 4:**
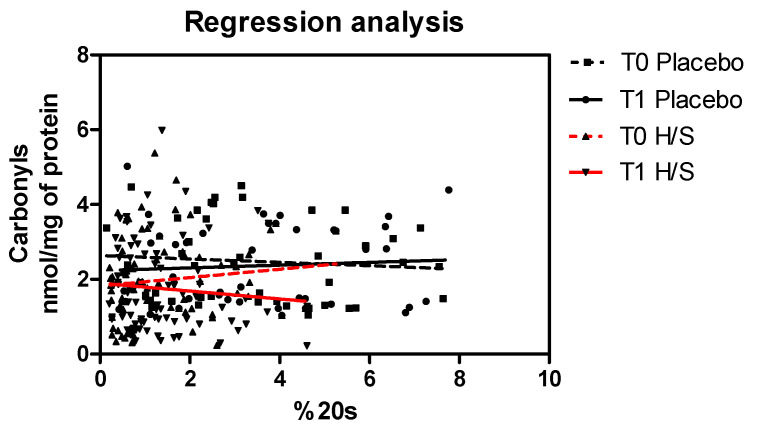
20S proteasome levels and levels of oxidized proteins: Regression analysis of measured biomarkers, as assessed in the placebo subgroup and the subgroup that received the composition (H/S), before (T0) and after (T1) the intervention. 20S proteasome levels were tested in PBMCs isolated from whole blood, and oxidized proteins were tested in isolated plasma.

Oxidative stress states can reflect an impaired antioxidant defense on multiple levels. To this end, we focused here not only on the depiction of oxidative stress status but also on its association with proteolytic mechanisms. Therefore, we investigated the interrelation of measured 20S proteasome levels and levels of oxidized proteins, which are the preferred substrates for enzymatic degradation by the proteasome. Regression analysis revealed an inverse correlation of proteasome content and levels of oxidized proteins after the intervention (T1 H/S), which was observed in the subgroup of volunteers who received the supplement (continuous red line in Figure 4), while the same effect was not observed in the placebo subgroup (T1 Placebo, continuous black line in Figure 4). In conclusion, the assessment of the interrelation between the two biomarkers represents the secondary outcome of the clinical in vivo study.

## 4. Discussion

Previous studies, also from our lab, established the antioxidant and antiaging effects of naturally derived compounds in various cell types and model organisms. For instance, flavonolignans found in *Linum usitatissimum* and *Silybum marianum* have attracted growing interest for their potent lifespan-extending, anti-aggregation and antioxidation properties in cell cultures and model organisms [37,38,39]. Similarly, for the polyphenolic compounds found in abundance in *Cynara scolymus* extract, and specifically for chlorogenic acid, numerous studies highlighted its ability to mitigate inflammation and oxidative stress by activating the Nrf2 pathway that regulates redox homeostasis [40,41]. Finally, another ingredient that was also used in the final composition was the mastic powder from *Pistacia lentiscus* L. var *chia*, an endemic tree grown on Chios Island in Greece. Mastic gum’s essential oil and pure compounds that the resinous extract contains in abundance, such as a-pinene and myrcene, have been shown to confer cytoprotective properties under oxidative stress conditions by upregulating a set of genes associated with antioxidant cellular response [42]. Moreover, the aqueous extract of mastic resin, as well as its major constituents, have been shown to exert antigenotoxic activity against DNA damage in human lymphocytes [43,44]. Given these data, herein, we dissected the detailed effects of the four natural ingredients combined in HS composition on cellular lifespan extension, proteostasis improvement, antioxidant capacity, maintenance of telomere integrity and preservation of physiological global DNA methylation levels.

Our data highlight the additive and synergistic effects of the identified combination comprising *Linum usitatissimum*, *Silybum marianum*, *Cynara scolymus* and *Pistacia lentiscus* extracts on the multiple aging biomarkers that were assayed. Essentially, treatment with the HS composition significantly increased the replicative lifespan of human fibroblasts. This increase in cellular lifespan positively correlated with the enhancement of CT-L proteasome activity and the expression levels of catalytic (β5), structural (α7) and regulatory (rpt6) 26S proteasome subunits, the prevention of telomere attrition and the maintenance of optimum DNA methylation levels. Additionally, the oxidative load of the cells, as measured by the total protein carbonyl levels, was significantly reduced. At a mechanistic level, the composition also activated two well-known pro-longevity factors that link nutrient sensing pathways and dietary restriction (DR) to lifespan extension: the transcription factor FoxO1 and the NAD+-dependent histone deacetylase (HDAC) SIRT1. FoxO transcription factors are evolutionarily conserved longevity regulators downstream of insulin and insulin-like growth factor signaling (IIS). When IIS is low, FoxO factors enter the nucleus to orchestrate the expression of pro-longevity genes, including the proteostasis network [45]. Recently, it has been shown that Foxo1 regulates the expression of the catalytic β5 subunit [13], and thus the increased FoxO1 transcriptional activity may account, at least partially, for the enhanced CT-L activity of cells treated with the composition. Simultaneously, SIRT1 plays a key role in lifespan and healthspan extension upon dietary restriction, regulates antioxidant cell response and contributes to telomere maintenance and genome integrity [25], and thus, the search for SIRT1 activators is one of the most extensive and robust topics of research [46]. The composition directly improved SIRT1 activity, and this possibly contributed to protection from telomere attrition and accumulation of oxidized proteins. Therefore, the composition protects from physiologically meaningful alterations that occur during senescence, with beneficial effects on cellular lifespan.

The antioxidant capacity of the composition was further confirmed in vivo, through the administration of the new supplement in the form of an oral capsule in healthy volunteers within the frame of a prospective, randomized, controlled 3-month trial. Factors such as sex, age and specific lifestyle habits, were shown to have an influence in the observed outcome. Specifically, female volunteers, participants aged 45–55 and with a BMI > 25, non-smokers and subjects who followed a balanced diet and exercise were the subgroups that exhibited the greater difference in levels of oxidatively modified proteins pre- and post-intervention. The permanent oxidative modifications of proteins are inescapable during cell metabolism in every living cell. Important evidence supports the fact that biomarkers such as oxidized plasma proteins are reliable in vivo indicators of organismal redox status [47,48]. In our study, we observed that even after a short-term administration of the composition, there was a significant decrease in oxidized plasma proteins. The fact that this reduction was not observed in the placebo subgroup, and no dietary and lifestyle changes during the intervention period were recorded for both subgroups, provides strong evidence that this change was attributed to the supplementation. Metabolomic analysis from the prospect of bioavailability could further explain mechanistically this important finding. However, to further assess the biological activity of the administered supplement, we investigated the interrelation between levels of oxidized proteins and measured 20S proteasome levels, as the latter represents the key player in the selective removal of oxidized proteins [49]. Previous studies have established in cellulo and in vivo the inverse relationship between proteasome levels and oxidized/carbonylated proteins in an effective proteostasis [5,6,7]. As assessed in cellulo, but as also reported in mammalian systems, proteasomes are highly responsive complexes that participate in multiple regulatory networks [50]. Analysis of the proteasome content was also performed before and after the intervention, but no quantitative differences were recorded. However, analysis in subjects who received the supplement revealed an inverse relationship of oxidized proteins’ levels and the proteasome after the intervention, while the same regression analysis in the placebo subgroup did not establish this negative correlation between the two biomarkers after the intervention. A negative correlation of proteasome levels and oxidized proteins is indicative of efficient proteolytic degradation of the latter and, therefore, this finding provides mechanistic evidence that the composition has a regulatory role in proteasomal proteolysis towards the decrease in oxidized proteins in vivo, as also depicted in cellulo.

Along with proteolytic mechanisms, biomarkers related to other hallmarks of aging, such as the relative telomere length and the global DNA methylation, were tested in vivo as well and did not exhibit profound changes after the intervention. Although telomere length is a reliable surrogate biomarker of senescence in simple cellular models, leucocyte telomere length in mammalian complex systems is apparently a very dynamic biomarker, also reflecting changes that are often transient, such as induction of immune responses by the different T-cell populations [51]. Moreover, for the assessment of changes in telomere length and also in the global DNA methylation profile, cells were treated with the composition during their whole lifespan. As such analogy could not be depicted in the short duration of the trial period, those biomarkers would be more appropriate targets of interventions which would be implemented for more extended periods of time, or even in longitudinal studies. Furthermore, and specifically regarding methylation levels, high tissue specificity has been observed in examined DNA from several somatic tissues, leading to uncertain conclusions about their optimal levels in mammals [52]. In addition, age and lifestyle are also critical factors that should be taken under consideration regarding normal or pathological levels of such biomarkers. Therefore, more work is needed to fully characterize these senescence hallmarks as robust biomarkers of aging in human studies [51,53].

Collectively, the antiaging, antioxidant and well-being properties of the examined composition are the result of a combined action against cellular oxidation, maintenance of the proteasome content and activity, the main secondary antioxidant mechanism of defense, maintenance of telomere length and preservation of optimum DNA methylation levels. An increasing line of evidence has emphasized the antioxidant properties of the selected natural compounds, through various in vitro methodological approaches [37,38,39,40,41,42,43,44,54,55,56]. However, to our knowledge, this is the first time that these compounds are combined into a complex that has additive/synergistic action and was tested on multiple hallmarks of aging, both in cellulo and in vivo. As mentioned, the advantage of aging biomarkers, which are representative of various hallmarks, was taken to define the biological action of the developed composition on human diploid fibroblasts, but also in healthy volunteers, and to explore the interaction of the combined extracts within this novel mixture. Different plant extracts possess different bioactive compounds with diverse antioxidant capacities. As a consequence, the total antioxidant capacity of different extract mixtures may be modified via synergistic, additive, or antagonistic interactions among these components, which may, in turn, alter their physiological impacts and may even deliver a pro-oxidant effect [57,58]. From a phytochemical perspective, the synergistic effect of different plant extracts may occur as a result of reactions such as regeneration of the stronger antioxidant by a weaker antioxidant, formation of stable intermolecular complexes between the antioxidants that exhibit higher antioxidant activity, formation of dimers and adducts or/and new phenolic products with greater antioxidant power than that of the single compounds, differences in solubility and phase distributions of antioxidants, or even unpredictable interactions among the examined compounds [59]. Several hypotheses have also been proposed for antagonistic interactions, such as antioxidant polymerization, which causes a decrease in their antioxidant properties or irreversible reactions of free antioxidant radicals, leading to their final disappearance. Moreover, there are factors that can influence the function of an antioxidant, transforming it into a pro-oxidant, namely the presence of metal ions, the concentration of the antioxidants in matrix environments and their redox potential [59,60]. It is worth noting that the synergistic or antagonistic effects are dependent on the type of antioxidants and their concentration [61]. The present study confirms the additive/synergistic interaction of the different constituents in the proposed quantities and ratios through a multidimensional biological evaluation. Given the complex nature of the aging process, it is unlikely that the most effective preventative antiaging therapy could be achieved by a single compound with a single target. In this study, we identified a combination of extracts that have the highest antioxidant capacity and an optimal beneficial effect on cellular lifespan by targeting different aging hallmarks. While all extracts displayed proteasome-activating properties and maintained optimal methylation, only *Linum usitatissimum* and *Cynara scolymus* could maintain telomere length. The observed additive/synergistic effects of the formulation can be attributed to the different molecular targets that are affected by the array of bioactive compounds. Metabolomic and biochemometric approaches, along with Big Data analyses, are needed to identify molecular targets of synergy and characterize the nature of their interactions, to shed more light on the biological activities of complex compound mixtures [58].

## 5. Conclusions

Overall, our data suggest that the different antiaging and antioxidant mechanisms examined act in a complex interplay that results in significant improvements, assessed for the first time in various hallmarks of aging in cellulo and in vivo. These observations are setting the ground for future studies to identify the long-term implications of such changes and focus on the underlying mechanisms of complementary action in antioxidant compound mixtures. We cannot rule out the possibility that the exerted pleiotropic beneficial effects may also employ other biological mechanisms that are still unidentified. Understanding the molecular processes of age-related health decline is of paramount importance for the study of aging pace, both as a target and as an outcome. Therefore, proper investigation of the effects of such nutraceutical combinations on aging biomarkers in the context of large interventional and longitudinal studies will further expand the arsenal of therapeutic strategies to delay aging and protect against age-onset pathologies.

## 6. Patent

Patent submitted to the Hellenic Industrial Property Organisation. e-filing number: 246-0004480602.

## Figures and Tables

**Table 1:** Composition of the oral capsule that was tested in cellulo and administered in the context of a clinical trial.

*Components*	mg/cap	%/cap	Active Ingredient (mg/cap)
***Linum usitatissimum* (Seed extract standardized to 40% SDG)**	100	21.98	40
***Silybum marianum* (Fruit extract standardized to 50% silymarin)**	50	10.99	25
***Cynara scolymus*** **(Leaf extract standardized to 5% chlorogenic acids)**	100	21.98	5
***Pistacia lentiscus* (resinous exudation) standardized to 12% isomasticadienonic acid)**	100	21.98	12
**Magnesium stearate**	10	2.20	
**Vegetarian Capsule No 0 (hydroxypropyl methylcellulose)**	95	20.88	
**Content weight**	360		
***Capsule total***	455		

## Data Availability

The data that support the findings of this study are available from the corresponding author upon request due to privacy/ethical restrictions.

## Supplementary Materials

The following are available online at www.mdpi.com/article/10.3390/antiox11030468/s1, Table S1: Primer sequences for Real-time PCR, Table S2: Technical details of each component of the composition, Figure S1: Assessment of short term effects of different extract combinations in primary human cell cultures, Figure S2: CONSORT 2010 Flow Diagram.
