# Supplementary material for: Antioxidant and Antiaging Properties of a Novel Synergistic Nutraceutical Complex: Readouts from an In Cellulo Study and an In Vivo Prospective, Randomized Trial
Source: Antioxidants (Basel). 2022 Feb 26;11(3):468. doi: 10.3390/antiox11030468 (PMC8944750; doi:10.3390/antiox11030468)
Supplement: Supplementary file 1 [file antioxidants-11-00468-s001.zip › antioxidants-1600431-supplementary.pdf]

Supplementary Table S1. Primer sequences for Real-time PCR.

Primer	Sequence	Target Template	Ascension number of target templates	Product Length
hGAPDH-R	5′-CATGGGTGGAATCATATTGGAA-3′	H. sapiens glyceraldehyde-3-phosphate dehydrogenase (GAPDH), transcript variants, mRNA	NM_001289745.3, NM_001289746.2 NM_002046.7	155
hGAPDH-F	5′-GAAGGTGAAGGTCGGAGT-3′			
hβ5-R	5′-CATCTCTGTAGGTGGCTTGGT-3′	H. sapiens proteasome 20S subunit beta 5 (PSMB5/β5), transcript variants, mRNA	NM_001144932.3 NM_002797.5 NM_001130725.1	119
hβ5-F	5′-AGGTTCTGGCTCTGTGTATGC-3′			
hα7-R	5′-ACAGCCCCAATAACCGTATG-3′	H. sapiens proteasome 20S subunit alpha 3 (PSMA3/α7), transcript variant 1, mRNA	NM_002788.4	104
hα7-F	5′-GACCTTTTGGCTGCAGTTTC-3′			
hRpt6-R	5′-GGATGCTTAACAGGCAGCTC-3′	Homo sapiens proteasome 26S subunit, ATPase 5 (PSMC5/RPT6), transcript variant 2, mRNA	NM_001199163.2 NM_002805.6 XM_024450841.1 XM_024450840.1	139
hRpt6-F	5′-TCCTGCCCAACAAGGTAGAC-3′			

Supplementary Table S2. Technical details of each component of the composition

	Linum usitatissimum L.	*Silybum marianum*	*Cynara scolymus L.*	*Pistacia Lentiscus L. var. Chia*
**Extraction solvent**	Ethanol/Water (30/70)	Water/Ethanol (20/80 V/V)	Water	N/A
**Preparation type**	Dry extract	Dry extract	Dry extract	Air-dry
**Part of plant used**	Seeds	Seeds	Leaves	Resin
**Description**	Fine powder	Powder	Powder	Powder
**Color**	Brown/yellowish-brown	Light yellow	Brownish	Whitish to light yellow
**Odor**	Neutral and slight sweet	Without characteristic odor	Characteristic	Characteristic of Chios Mastiha aroma.
**Taste**	Characteristic	Characteristic	Characteristic	Characteristic
**Assay:**	Lignans (as SDG) > 20% (HPLC-UV)	Min. 50% Silymarin (HPLC)	Caffeolquinics acids expressed in chlorogenic acid 5-7.2% (HPLC)	Isomasticadienonic acid 12-15% (HPLC)

A)

Extract Combination	Linum usitatissimum (in µg/ml)	Silybum marianum (in µg/ml)	Cynara scolymus (in µg/ml)	Pistacia lentiscus (% v/v)
**1**	20	10	20	0,05
**2**	10	5	10	0,025
**3**	5	2,5	5	0,0125
**4**	20	2,5	5	0,0125
**5**	5	10	5	0,0125
**6**	5	2,5	20	0,0125
**7**	5	2,5	5	0,1
**8**	10	10	10	0,05

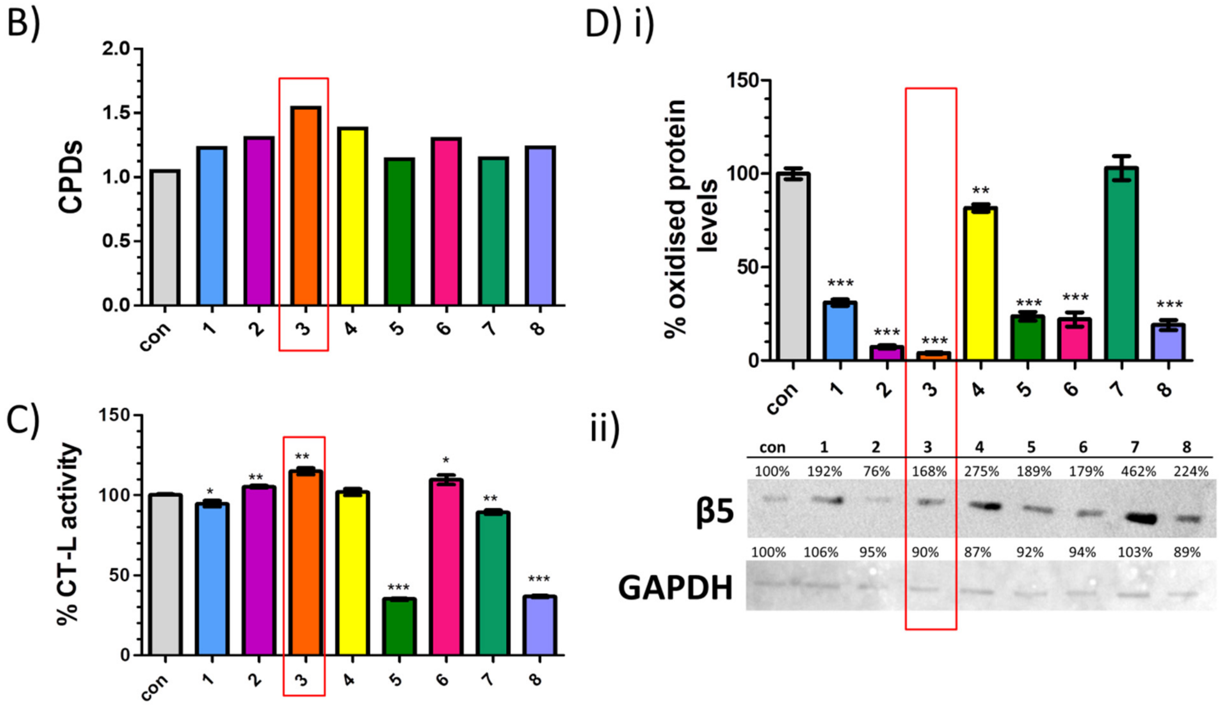

Supplementary Figure S1: Assessment of short term effects of different extract combinations in primary human cell cultures. (**A**) Table indicating the ingredients of each tested extract combination (1–8). (**B**) Number of cumulative population doublings (CPDs), (**C**) % CT-L activities, and (**D**) (i) analysis of carbonylated proteins and (ii) immunoblot analysis of the catalytic proteasome β5 subunit in total cell extracts from human fibroblasts treated with extracts combinations or the solvent (control), for 72h. GAPDH was used as control for equal protein loading. The signal in control cells was arbitrarily set to 100%. ***: $p < 0.001$, **: $p < 0.01$, *: $p < 0.05$.

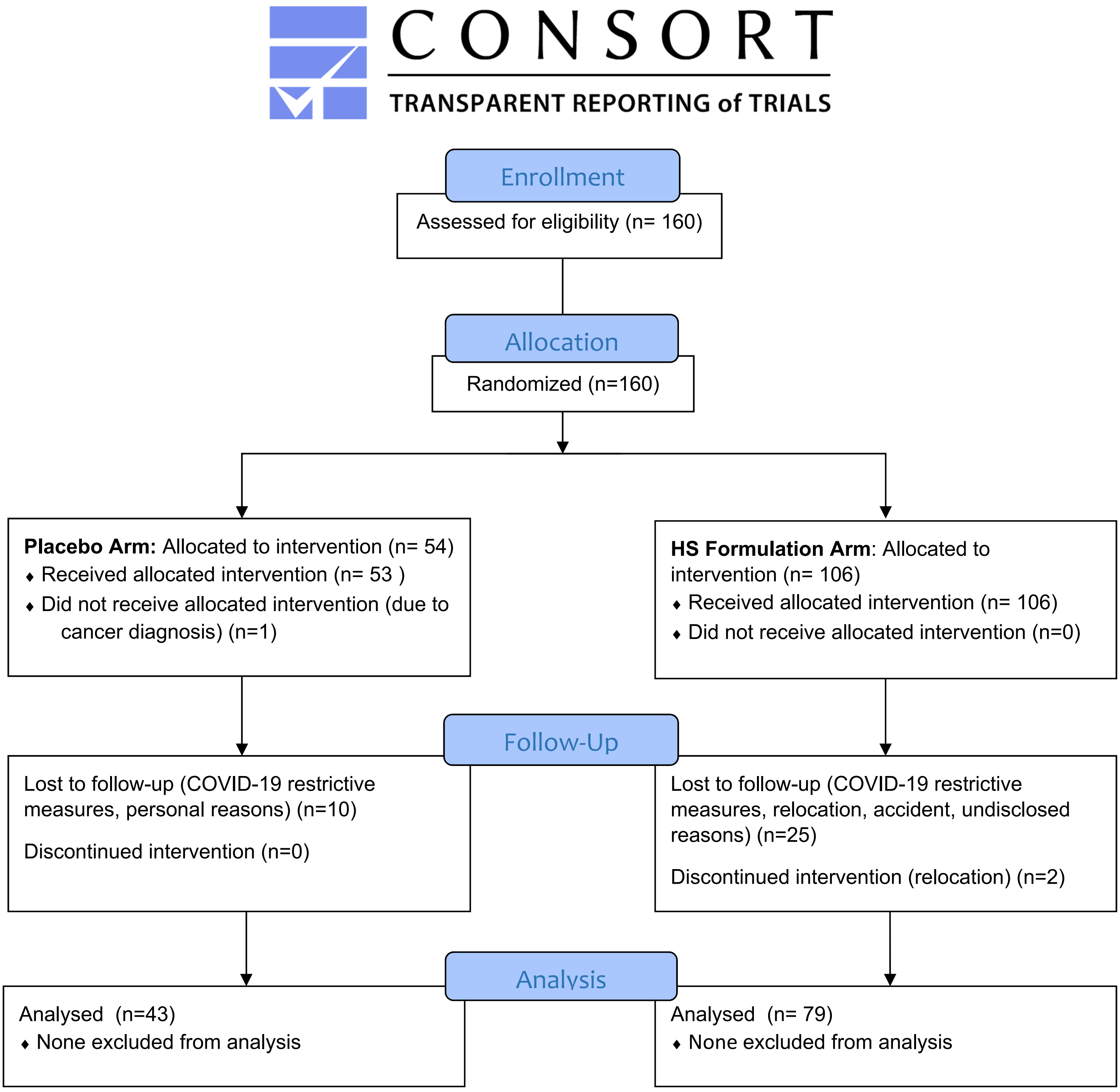

Supplementary Figure S2: CONSORT 2010 Flow Diagram.
